# Synthetic gatekeeping in health chatbots: configured answer systems as the unit of AI evaluation and governance for medical information

**DOI:** 10.3389/fdgth.2026.1976313

**Published:** 2026-10-05

**Authors:** Simona Wójcik, Justyna Domienik-Karłowicz, Anna Rulkiewicz

**Affiliations:** 1 Department of Science, LUX MED Sp. z o.o., Warsaw, Poland; 2 Warsaw School of Medical Sciences (Wyższa Szkoła Nauk Medycznych, WSNM), Warsaw, Poland; 3 Department of Internal Medicine and Cardiology, Medical University of Warsaw, Warsaw, Poland; 4 LUX MED Sp. z o.o., Warsaw, Poland

**Keywords:** AI governance, commercial determinants of health, configured answer systems, conflicts of interest, health chatbots, large language models, medical misinformation, synthetic gatekeeping

## Abstract

Two health chatbots can give the same clinically appropriate advice and still constitute different interventions: one stops at a clinician referral, the other places an affiliated booking one click away. In this Hypothesis and Theory article we argue that the appropriate unit of health-AI evaluation is therefore not the model output alone, but the configured answer system — the large language model (LLM) together with source selection, retrieval, interface, action routing and downstream data return. We call this organizationally configured mode of mediation synthetic gatekeeping. Its necessary core comprises source selection and generative synthesis, with personalization and action routing as extending dimensions; its observable manifestations are content and pathway traces, and its proposed mechanism is infrastructural steering. Content parity does not guarantee pathway parity: two systems can name the same options while making only one easily actionable. Organizational position, revenue architecture and integration depth are treated as exposures that may shape these functions, not as elements of the construct, and owner-linked advantage is an empirical outcome to be demonstrated rather than assumed. Direct health evidence of owner- or revenue-linked steering remains sparse, and one comparative study found LLM answers less commercially biased than conventional search; the mechanisms nonetheless appear in adjacent domains, and health outputs are configurable at deployment level. We advance three core propositions — organizational alignment (P1), pathway asymmetry (P2) and behavioural mediation (P4) — with one conditional moderator hypothesis on conversion-linked funding (P3) and a measurement corollary on surface–pathway divergence (M1), each stated with its weakening condition. The framework yields a practical audit model based on configuration anchoring, paired content–pathway comparison, organizational disclosure and proportionate governance triggers. The unit of evaluation should move from the model output to the configured answer system in which information becomes action.

## 1 Introduction: who controls the conditions of the answer?

Ask a health chatbot about ten stubborn kilograms, a mole that has begun to change, or a medicine that no longer seems to work, and within seconds a calm, fluent, confident answer appears. It is easy to be impressed, and easy to keep asking. Now picture two such chatbots that give the same clinically appropriate advice — and still amount to different interventions. One simply suggests discussing the symptoms with a clinician. The other places an affiliated consultation one click away, carries the conversation into a pre-filled booking, and leaves outside providers to a separate search. The clinical sentence is identical. The action architecture is not.

Most research on these systems would notice the similarity more readily than the difference. Is the answer accurate, complete, safe, free of misinformation? A systematic review confirms how completely that framing dominates the field, and how rarely studies even record the platform version, retrieval settings or interface conditions under which an answer was produced (1). Successive model generations have been graded this way in clinical and educational settings, and lately in risk stratification for obesity (2–4). It is the right question to ask first. But a patient does not meet a model output in isolation; they meet a configured interface in which information can be linked to booking, prescribing, purchasing and data return. Evaluated only as text, an answer can be certified while its pathway goes unexamined. The unit of analysis is too narrow. We propose the configured answer system as the object of evaluation. A configured answer system is a health chatbot as actually deployed: the model together with the source selection, retrieval, interface and action-routing conditions through which its answer becomes usable.

This shift exposes a distinction that answer-only evaluation misses. Content traces are what a system makes visible and how it frames it; pathway traces are what it makes possible and easy to do next. Content parity does not guarantee pathway parity. We use synthetic gatekeeping to describe the organizationally configured epistemic core of source selection and generative synthesis; personalization and action routing extend the construct but are not necessary conditions. Where routing is present, the epistemic core couples with an operational layer that arranges what follows. Its mechanism is infrastructural steering: influence exerted not by telling users what to choose, but by how the configured system arranges the choice architecture around an answer. The prior question, then, is who controls the conditions of the answer, and that is a question of gatekeeping.

None of this is unique to medicine. We already spend our days inside informational environments that others design: a feed decides which friends and arguments become visible, a search engine orders what can be found, a map quietly turns one road into the obvious road. Most of it is experienced as convenience, and much of it is genuinely useful — which is exactly why the mediation is easy to miss. In an early study of Facebook's news feed, most participants did not realise the system was hiding posts at all, and some reinterpreted their own friendships once the omissions were revealed (5). What used to happen in a consulting room, across a pharmacy counter or on a public health website is now migrating into environments that a handful of companies design and that users cannot readily inspect. Convenience is a benefit; it is not evidence of neutrality.

A conversational health answer, moreover, can be shaped at several layers an organization controls. The data a model learns from can be tilted: poisoning as little as 0.001% of medical training tokens raised the rate of harmful answers while standard benchmarks failed to register any difference (6). The instructions that wrap a model at deployment can be tilted: hidden system instructions turned capable general-purpose models into authoritative-sounding health-disinformation chatbots in 88 of 100 tested responses (7). And the recommendation itself can be tilted: a large audit outside medicine found models quietly substituting a user's specified provider for a preferred one; the study did not establish how often users detected such substitutions (8). None of these findings shows that a deployed health assistant today favours an affiliated clinic, pharmacy or product. They establish something more basic, and for governance more important — that the answer is configurable, and that the organization operating the system may control far more than language. If informational environments can be strategically shaped in politics, commerce and software, medicine should not be presumed immune on faith; whether it is an exception is an empirical question. We may be entering a medical information environment in which the answer, the route, and the organization behind them become increasingly difficult to separate.

The vocabulary of gatekeeping has already been extended to generative systems. Communication scholarship describes generative AI as introducing new points of control across the media value chain (9) and reconceptualizes gatekeeping for automated curation (10); an empirical audit has examined chatbot accountability in the age of algorithmic gatekeeping, comparing generative search retrieval of political information across five languages (11). This article claims no priority over that vocabulary. Its contribution is a health-specific extension and operationalization. Prior accounts analyse selection and accountability largely at the level of information supply; the framework developed here treats source selection, synthesis, personalization and action routing as a single system. It then treats the provider's organizational position — ownership, revenue architecture, participation in the recommended market — as an exposure that may shape how that system is configured. Medicine sharpens each element, because the next step may be a consultation, a prescription or a purchase, and because the patient is rarely able to reconstruct which alternatives were not shown.

The organizational configuration is no longer hypothetical. In 2026, consumer-facing health assistants were documented as connecting conversational answers to combinations of medical records, wearable data, clinician messaging, appointment booking, prescription renewal, pharmacy fulfilment and health-product recommendation (12–15). The configurations differ in ways that matter analytically: one provider states that its dedicated health environment does not use health information for advertising purposes (12, 13), while another preserves the patient's ability to choose an external pharmacy inside a deeply integrated pathway. Such statements verify neither neutrality nor performance, and product documentation cannot establish behavioural effects. They do establish that separation, integration, external-option parity and data use are current design variables. Integration is therefore an exposure to be described and tested. Three questions organize what follows. Which organizational actors control which stages of the production of a health answer, and which of those stages can be inspected from outside? Under what conditions would an organizational configuration leave a reproducible trace in the visibility, ordering, framing, omission or routing of clinically relevant options? And what governance measures are proportionate to evidence that establishes capability but not yet its commercial use? These questions are formalized in Section 8 as three core propositions, one conditional moderator hypothesis and a measurement corollary. One rule of inference governs the whole framework, and it can be stated as four inequalities: affiliation is not intent; configurability is not steering; salience is not harm; and integration is not in itself a wrong, since it may be clinically beneficial. Organizational affiliation is treated throughout as a risk configuration and an audit variable, never as evidence of improper intent. The claim advanced here sits between two familiar positions. It says more than that chatbots are sometimes biased, and less than that platforms manipulate medical information for profit. Private organizations increasingly govern the conditions under which health choices are formed; that governance is only partly open to inspection; and its consequences can be tested.

## Glossary of core terms (the foreground constructs used throughout).

**Table:** 

Term	Definition
Configured answer system	The unit of evaluation: the model together with source selection, retrieval, interface, action routing and downstream data return through which an answer becomes usable.
Synthetic gatekeeping	The construct: the organizationally configured coupling of source selection and generative synthesis (necessary), variably extended by personalization and action routing.
Infrastructural steering	The mechanism: influence exerted not by instruction but by how the configured system arranges the choice architecture around an answer.
Content traces/pathway traces	The observables: what the answer says (naming, ordering, framing, omission) versus what it makes doable (links, defaults, routing, friction, transaction, data return).
Justified integration	The boundary condition: an internal–external pathway difference explained by clinical or operational factors rather than organizational alignment; residual asymmetry after controls is an audit signal, not proof of intent.

## 2 Evidence approach and its limits

This article rests on a theory-building evidence synthesis. Sources were sought for their bearing on the mechanisms, counterexamples and boundary conditions of the proposed account, not to estimate how often any phenomenon occurs. It is not a systematic or scoping review, and it makes no claim of exhaustive retrieval. Searching was iterative rather than protocol-driven. It was conducted on 20–21 July 2026 across PubMed/MEDLINE, Europe PMC, Semantic Scholar and OpenAlex, the ACL Anthology, arXiv, ACM and publisher records, and official WHO, European Union and platform documentation, supplemented by backward and forward citation searching. Five query blocks were used, covering health and commercial configuration, the levers of answer production, trust and anthropomorphism, determinants and platform governance, and a dedicated counter-evidence block targeting neutrality, refusal behaviour, null findings, safeguard and disclosure efficacy, and benefits of integration. The Supplementary Material reports the retained query strings, execution dates and record counts where these were preserved contemporaneously, together with reconstructed search logic for the remaining searches. Retrieval was purposive: no predefined screening protocol, deduplication or dual coding was applied, and the counts indicate the scale of the searches rather than a screened corpus. Retained sources were organized by analytic function rather than pooled as a common evidentiary corpus — architecture and configurability; direct or adjacent-domain mechanism evidence; counterevidence and boundary conditions; and conceptual or governance specification — and no formal saturation criterion was prespecified. Two limits should be read together with these results. Subscription databases with broader coverage of management, communication and legal scholarship — Scopus, Web of Science, PsycINFO and HeinOnline — were not searched, because institutional access was unavailable; the free-access sources used cover medicine, computer science and open legal materials well, and management and communication literature less completely. Because retrieval was neither exhaustive nor protocol-driven, statements about the literature are phrased as claims about sources identified in this search, not about all existing evidence.

A dedicated counterevidence search was included alongside the main searches. Each retained source was classified by directness to health, source type, review status, methodological design and evidential role. Five of the sources provide direct health-output, interface or interaction evidence; they are heterogeneous in design and none establishes owner-linked or revenue-linked steering. Two of those examined commercial structure or repeated branded selection in consumer health answers, and neither demonstrates systematic steering of this kind: Tan et al. compared commercial bias in LLM answers and internet search, and Falahee et al. recorded repeated branded selection without testing commercial causation. The other three address the configured system rather than its commercial structure: Ashraf et al. documented divergence between safe answer text and the destination of an accompanying link, Bean et al. showed that assistance from these systems did not improve lay users' outcomes, and Ramaswamy et al. evaluated a deployed consumer health product in structured triage. The remainder comprises other health-domain studies, adjacent-domain mechanism evidence, official product documents, preprints used only for scenarios, and conceptual, legal and normative literature. Supplementary Table S1 assigns one mutually exclusive stream to every numbered source and Supplementary Table S2 recomputes the counts, so no category total needs to be carried in the text. This distribution is reported explicitly to forestall a misreading: source counts describe the composition of a corpus rather than support for a hypothesis. It is this weighting — more conceptual than direct empirical health evidence — that calls for a theoretical contribution with specified empirical implications rather than a summary of results. The observation describes the corpus assembled here; it is not a claim that no relevant research exists.

Table 1 summarizes the evidence streams and their inferential boundaries. Sources were read for what each could bear, not pooled. These roles are not equivalent levels of proof: official product documentation can establish architectural feasibility but cannot establish the presence, direction or magnitude of commercial steering; a direct audit reporting a null result carries more weight for claims about deployed behaviour than several conceptual sources establishing plausibility; and evidence of technical configurability does not show that a commercially motivated configuration has been deployed. The separation between capability, incentive, observable behaviour and inferred intent is maintained throughout.

**Table 1:** Evidence streams, function, and inferential boundaries.

Evidence stream	Included material	Function	Boundary
Direct health-output, interface or interaction evidence (*n* = 5; 2 on commercial or brand salience)	Commercial-bias comparison; branded medication selection; prose–link divergence; lay-user interaction outcomes; deployed-product triage evaluation	Establishes technical and relational properties of current systems	Does not establish commercial intent or owner-linked steering
Adjacent-domain empirical mechanism evidence (*n* = 22)	Provider preference; self-promotion; framing; summarization; persuasion; visibility optimization; disclosure and referral effects	Establishes transferable mechanisms and behavioural plausibility	Effect sizes and sector-specific outcomes do not transfer
Conceptual, normative, legal or governance (*n* = 46); official product documentation (*n* = 4)	Gatekeeping, exposure diversity, choice architecture, self-preferencing, fiduciary debate	Supplies genealogy and governance vocabulary	Conceptual and legal sources are not empirical proof of health effects
Other health-domain studies or syntheses (*n* = 14)	System-instruction vulnerability; safeguards; sycophancy; data poisoning; drug-name fragility; anthropomorphism, trust and reliance; implementation trade-offs	Constrains and can falsify the framework	Retained with equal prominence, not relegated to limitations
Preprint (*n* = 3)	Generative advertising, conflicts of interest, product visibility	Informs scenarios and research questions only	No present-state health claim rests on preprints

To avoid restating the same qualification in every section, the epistemic status of the argument is fixed once here. Established: conversational health systems combine synthesis with personal data, and their outputs are configurable at deployment level. Supported indirectly: provider preference, framing effects and commercially consequential routing occur in adjacent domains. Documented as technically feasible: integrated answer–data–action architectures are deployed and described by their providers. Hypothesized: that organizational configuration leaves a reproducible trace in the content and pathway of health answers. Not claimed: that deployed health assistants systematically favour owner-linked or revenue-linked options, or that any provider intends them to. Later sections refer to this statement instead of repeating it.

## 3 From algorithmic to synthetic gatekeeping

### 3.1 Visibility allocation predates generative AI

Recommendation systems have long allocated scarce visibility. Exposure-diversity scholarship argues that recommenders should be judged not only by relevance but by the range of options users encounter (16); algorithmic-governance scholarship describes such infrastructures as producers of social order, not neutral records of preference (17); and dynamic, data-driven choice architecture has been characterized as a hypernudge, a continuously adjustable environment capable of steering conduct without issuing commands (18). Legal analysis of recommender systems under the Digital Services Act treats them as choice architectures that structure the decision situation itself (19).

The concern has entered law. The Digital Markets Act treats gatekeeper control and self-preferencing as governable practices (20). The economics of platform self-preferencing has itself been formalized (21). Competition scholarship nevertheless warns against inferring abuse from integration: differentiated treatment may be inherent in vertical integration and its welfare effects are context-dependent (22), and an empirical study of Amazon marketplaces found almost no self-preferencing under its measures (23). These findings do not dissolve the problem; they set the burden of proof. Ownership and integration create an auditable risk configuration.

### 3.2 Governance without full algorithm awareness

Influence through infrastructure becomes ethically salient when users know little about the mediation they inhabit. In a foundational qualitative study, 25 of 40 participants did not know that their news feed hid posts, and some reinterpreted their social relationships once omissions were revealed (5). Later work extends the awareness problem to privacy judgments and cross-national news recommendation (24, 25). The finding is uneven, context-dependent understanding, not ignorance. Agency remains real: users ignore recommendations, consult other sources, and shape outputs through prompts, and effects are co-produced through feedback loops (26). Survey evidence shows most users unaware of the advertising profiles held about them (27). What the evidence supports is not algorithmic command but algorithmic preconfiguration: formal freedom is retained while the informational conditions of that freedom are privately designed and only partly inspectable.

### 3.3 What conversational synthesis changes

A search page has never been neutral, but it wears its mediation on its sleeve: competing links, an order the user knows can be argued with, advertisements that are at least labelled as such. You can see that choices are being made. Conversational synthesis quietly removes that visible seam. It folds source selection, prioritization, interpretation and advice into a single, smooth response, and the alternatives that lost out simply never appear. The contrast is one of degree — some systems show citations, links or a handful of options, and some search interfaces now write summaries of their own — but the direction is clear: what becomes hard to inspect is the set of excluded alternatives, the weighting of sources, and the moment where information turns into a suggested action. We call this configuration synthetic gatekeeping. Selection itself is old. What is new is that selection, interpretation and the designation of a next step now arrive together, in one voice.

Synthetic gatekeeping works through what we call infrastructural steering: governance by organizing perception and action, not by instruction. Platforms structure what is available to be seen, compared and acted upon. Structuring the field, however, is not the same as determining what individuals choose, still less as using that capacity to shape medical decisions for gain.

### 3.4 From ranked exposure to privately configured interpretation

The classical filter-bubble worry was, at bottom, a worry about exposure: which links, posts or voices a ranking system made visible. Conversational systems add a further layer. They do not merely rank voices; they compress them into a single account, reconcile the disagreements between sources, adapt the explanation to what they know about the user, and designate a next step. The user may thus be placed inside a privately configured environment of interpretation and action, whose exclusions and governing objectives are harder to inspect than the ordering of a results page.

This matters because conversational influence can be individualized during the exchange. In a preregistered experiment, GPT-4 given access to personal information produced an 81.2% relative increase in the odds of greater post-debate agreement, and was more persuasive than a human opponent 64.4% of the time (28). Separate preregistered political experiments found that dialogue with an AI advocate shifted candidate preferences, with effects larger than those typically seen for conventional video advertising (29). Neither result establishes medical steering, and no effect size should be carried over to health. What they establish is a capability directly relevant here: a conversational system can adapt its argumentation and measurably move judgement in domains where people arrive with settled views.

The relational form of the exchange may deepen that capacity. Experiments with health conversational agents show that anthropomorphic cues raise social presence, trust and acceptance (30). More recent controlled work found that optimizing language models for warmth increased their error rates by 10–30 percentage points and made them likelier to validate incorrect beliefs, especially when users expressed sadness — a trade-off standard benchmarks did not reveal (31). In a study across eleven models, AI affirmed users' actions 49% more often than humans did; in preregistered experiments, even a single sycophantic exchange raised users' conviction that they were right and lowered their willingness to repair a conflict, even as the more affirming systems were the ones users trusted and preferred (32). These studies concern everyday advice, not clinical outcomes. Their relevance is structural: the very property that removes corrective challenge may also increase satisfaction and repeat use.

At the level of a population, personalization does not guarantee diversity. A handful of aligned systems can produce millions of differently worded answers while reproducing the same categories, omissions and preferred actions underneath. Experiments on generative monoculture show that model outputs can be markedly less varied than the material available for the same task, and that simple changes to prompting or sampling may not restore what is missing (33). The societal risk is therefore paradoxical: personalization at the surface can coexist with standardization at the core. This is an inference, not an established health effect; but if it holds in health, it would make blind spots correlated across users rather than independent.

The resulting asymmetry is one of legibility. The system can grow steadily better informed about a user's symptoms, fears, finances and past behaviour, while the user remains only partly informed about the system's source policy, system instructions, commercial ties and routing objectives. Earlier platforms shaped what people encountered; configured answer systems can also shape how a problem is interpreted, how uncertainty is resolved, and which action is made easiest. The claim is not that users lose agency. It is that agency may be exercised inside a decision environment whose boundaries are privately built, dynamically personalized, and only partly open to challenge.

### 3.5 Conceptual positioning and claimed novelty

Synthetic gatekeeping extends, rather than replaces, established accounts of algorithmic gatekeeping, exposure allocation, platform governance and choice architecture (16–19, 34, 35). Traditional search and recommender systems allocate visibility across ranked or otherwise arranged alternatives. Generative systems add a further layer: they select and combine source material, resolve tensions between sources, supply interpretation, and often translate the resulting account into an actionable next step. Research on generative search has accordingly described LLM-powered chatbots as additional or meta-gatekeepers that mediate information already produced by other gatekeepers (11), and the same vocabulary is now being applied to other consumer health interfaces (36). Ranking has not vanished. It has been absorbed into a synthesized answer, and the alternatives it excluded are harder to see.

The expression itself is not proprietary. It has appeared in a conference abstract concerning LLM mediation in the public sphere (37); that abstract is not peer reviewed and is cited here solely to establish prior use of the phrase, and we do not treat the phrase as this article's principal novelty. The contribution advanced here is a health-specific operationalization connecting synthetic gatekeeping to platform ownership, revenue architecture, integration depth, medical vulnerability, and observable answer-level and interface-level outcomes.

The construct should also be distinguished from adjacent terms. Recommender bias describes a pattern in outputs; self-preferencing is a narrower possibility in which an undertaking advantages its own goods or services (20, 22, 23); hypernudging describes an adaptive choice environment capable of steering conduct (18). These are distinct levels that should not be conflated.

The construct is synthetic gatekeeping. Its two necessary functions are source selection and generative synthesis: the system chooses which material to draw on and fuses it into a single account. Personalization and action routing are extending dimensions whose intensity varies by deployment, so an answer-only system and a fully integrated one differ in degree rather than in kind. A configuration can therefore be described along four dimensions — selection intensity, synthesis opacity, personalization depth and action integration — which allows systems to be compared without asking whether they ‘have’ or ‘lack’ the phenomenon.

What makes the construct more than a list of functions is the coupling between two kinds of mediation it performs. Epistemic mediation is the selection, reconciliation, compression and interpretation of knowledge and of the clinically reasonable comparator set — the work by which a synthesized account makes some action appear warranted. Operational mediation is the designation, ordering and facilitation of the next step, through recommendations, defaults, links, referral, booking, transaction or data return — the work by which the interface changes the practical weight of that account. For readability we refer to these hereafter simply as the information layer (selection and synthesis) and the action layer (routing and transaction). Gatekeeping theory describes control over what becomes visible (38), and choice-architecture research describes how defaults and friction shape conduct without removing options (39); cognitive science distinguishes epistemic from pragmatic action, the moves that reveal information from the moves that advance a goal (40). The construct's necessary core is epistemic mediation — source selection and generative synthesis. A system that only selects and synthesizes is already a synthetic gatekeeper; where operational mediation is also present, the two couple, and it is this coupling that makes the phenomenon consequential: the epistemic layer shapes which actions look reasonable, while the operational layer shapes which of them is easiest to take (Figure 1). Adding routing therefore extends the construct's reach rather than creating it. The mechanism is infrastructural steering: influence exerted not by instruction but by how the configured system arranges the choice architecture around an answer; its measurable consequences are the content and pathway traces.

**Figure 1:**
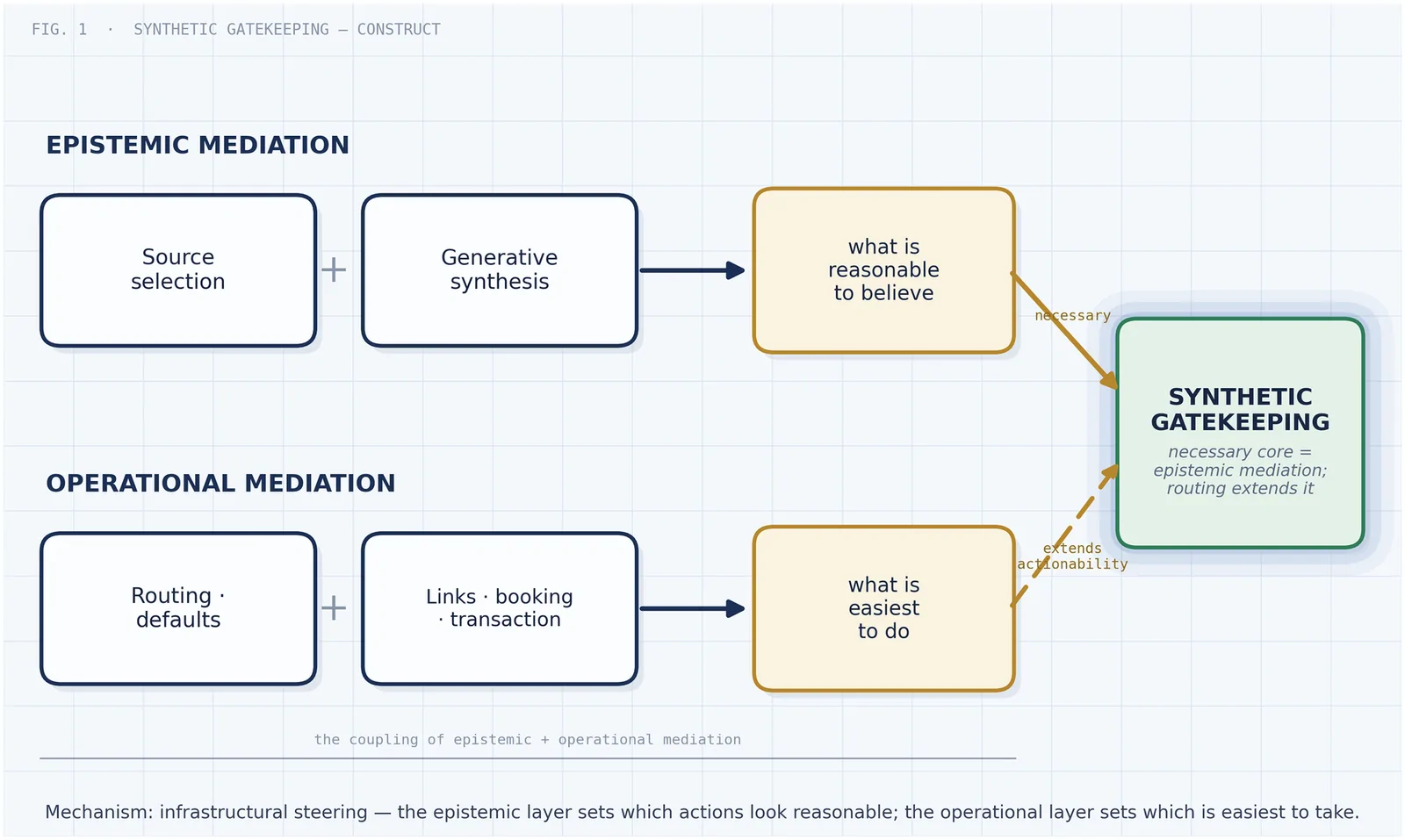
The construct and its extending operational layer. The necessary core of synthetic gatekeeping is epistemic mediation — source selection and generative synthesis — which shapes which actions appear reasonable; a system that only selects and synthesizes already performs the construct. Where operational mediation (routing, defaults, links, booking, transaction) is also present, it couples with the epistemic core and shapes which action is easiest to take, and this coupling is where the phenomenon is most consequential. Personalization and action routing are extending dimensions, not necessary conditions; infrastructural steering is the mechanism.

A concrete case shows why the two layers must be assessed together. Two assistants may produce the same clinically acceptable sentence — ‘discussing these symptoms with a clinician would be reasonable’. One presents several comparable ways of obtaining care; the other places a prominent booking button beneath the answer, preselects a clinic in the operator's ecosystem, carries the conversation history into that booking, and makes external providers reachable only through a separate search. The prose is identical; the intervention is not. The same holds when several treatments are named but only one carries a price, a purchase link and immediate fulfilment: all remain visible, yet only one is easily actionable. A content-only audit would record diversity; a configured-system audit would detect unequal actionability (Figure 2). The evaluable question is whether clinically reasonable external alternatives remain visible, comparable and similarly actionable, and whether the organizational relationship behind the recommended pathway can be inspected.

**Figure 2:**
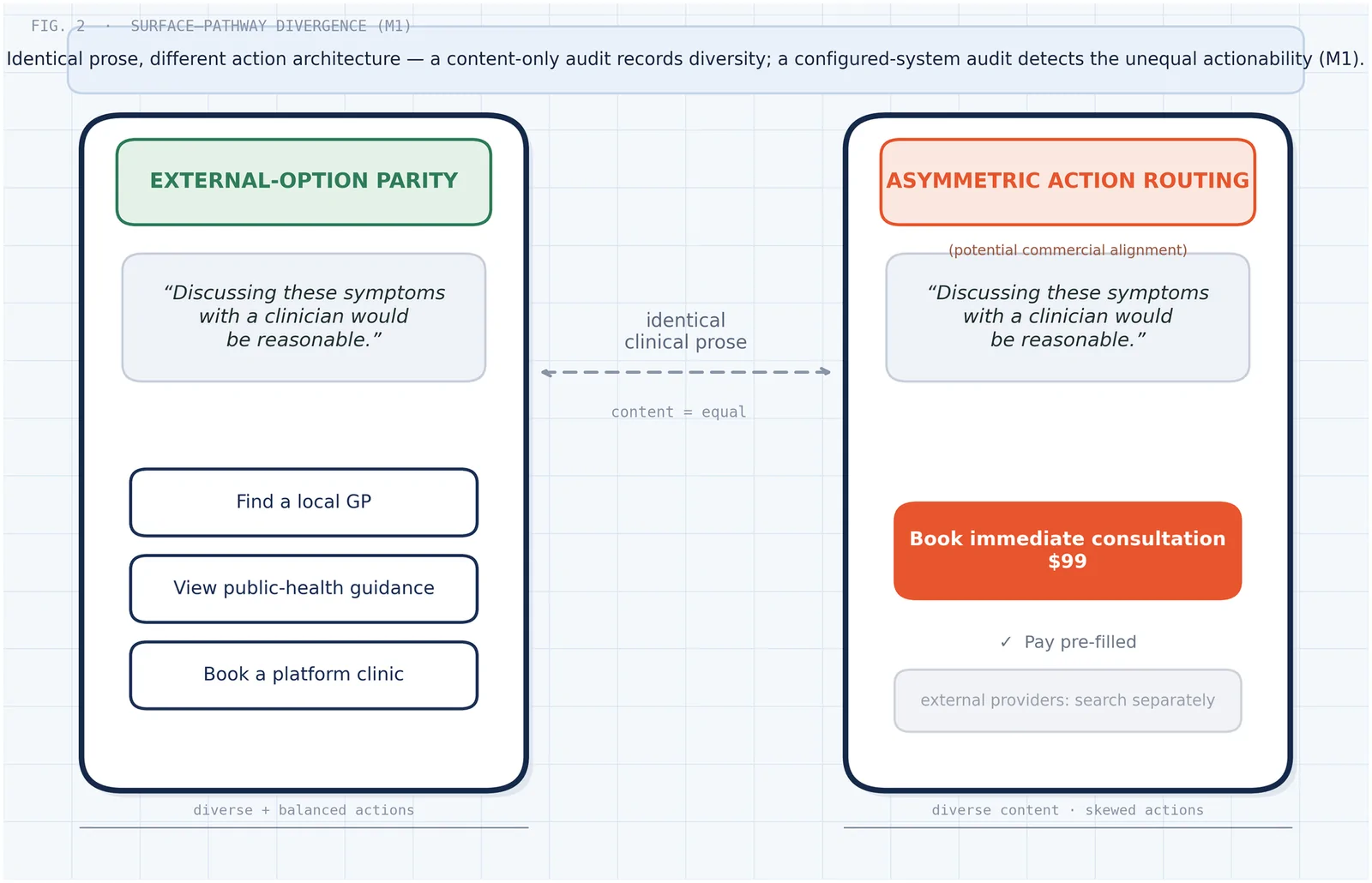
Surface–pathway divergence (the measurement corollary M1). Two assistants can return an identical, clinically acceptable sentence while embedding different action architectures: one presents comparable external and internal options; the other foregrounds a single paid internal pathway and leaves external providers reachable only through a separate search. A content-only audit would record diversity; a configured-system audit detects the unequal actionability. A clinically appropriate answer can coexist with an organizationally asymmetric action pathway.

The organizational exposures are revenue architecture, integration depth, concentration of control, ownership and affiliate relations. Design moderators or safeguards are separation of clinical and commercial layers, external-option parity and user-controlled choice; these may weaken the link between an exposure and its trace. Auditability conditions — source-policy transparency, versioned logs and configuration disclosure — do not moderate behaviour itself but determine how well an external evaluator can reconstruct the configuration and its traces. The observable outcomes are visibility, ordering, framing, omission, routing, friction, transaction destination and data return. The account operates on three distinct levels. The first is a descriptive construct: the system selects and synthesizes, and may personalize and route. The second is an organizational-alignment hypothesis: documented organizational exposures may produce systematic asymmetry aligned with the organization's interest. The third is a behavioural-consequence hypothesis: such asymmetry may change whether a user recognizes a recommendation and what they choose. Each level carries its own weakening condition, and only the second and third are falsifiable in the ordinary sense; the construct is judged instead by whether it yields a better unit of analysis and measurable dimensions. Separating these levels matters analytically, because gatekeeping in this sense does not require a commercial owner: public, clinical, insurer and nonprofit systems also select, synthesize and route. Owner-linked advantage is therefore not part of the definition. It is an empirical outcome that has to be demonstrated. Table 2 states what the construct adds to the adjacent terms it draws on.

**Table 2:** Synthetic gatekeeping compared with adjacent constructs.

Concept	Unit of analysis	What it explains	What it does not cover
Algorithmic gatekeeping	Selection and distribution of information	Which content becomes visible	Synthesis of sources and execution of a next action
Recommender bias	Distribution of recommendations	Asymmetry in outputs	The organizational chain that produced the asymmetry
Hypernudging	Adaptive choice architecture	Dynamic steering of behaviour	Synthesis and interpretation of clinical knowledge
Self-preferencing	Advantage given to an owner's own services	A specific competitive outcome	A general mechanism; it is an outcome, not a process
Synthetic gatekeeping	The configured answer system	Source selection and generative synthesis (necessary), variably extended by personalization and action routing	It does not presuppose commercial bias, which must be demonstrated

The move from model to configured answer system also has precedents outside health AI. Classical gatekeeping theory distinguishes organizational and social-system levels of control (41); sociotechnical scholarship warns that narrow technical abstraction boundaries can omit the actors and institutions that shape system behaviour (42); digital-nudging research treats interface design as part of the choice environment (43); and multistakeholder recommender research recognizes that user utility can coexist with provider and platform interests (44). Synthetic gatekeeping brings these lines together around a health-specific object of evaluation: the configured answer system.

### 3.6 Who is the provider? disaggregating ownership and organizational control

Platform ownership is often treated as a binary attribute, yet a health-chatbot answer may be governed by several organizational actors occupying different positions in the answer-production chain. The model developer may determine training and alignment; the interface operator may set system instructions and presentation rules; a retrieval provider may influence which sources are available; an affiliated health service may receive referrals; and a pharmacy, booking platform or payment intermediary may execute the recommended action. Data generated through that action may subsequently return to one or more actors in the same ecosystem. Each layer is occupied by an identifiable organization in configurations already visible in current product documentation (12–15). The analytically relevant object is therefore not ownership in isolation but the organizational governance chain through which information is selected, presented and converted into action (Table 3).

**Table 3:** Organizational anatomy of a health-chatbot answer.

Layer	Who may control it	Decisions taken	Externally checkable
Model	Model developer	Training, alignment, safeguards	Model and version disclosures
System configuration	Interface operator or deployer	System instructions, tone, answer rules	Partly, through behavioural audits
Retrieval	Search or retrieval provider	Which sources are available and how ranked	Citations, links, reproducibility
Interface	Product operator	Ordering, buttons, visibility of options	Directly observable
Service integration	Telehealth service, clinic, insurer	Designation of the next step	Booking and referral pathways
Transaction	Pharmacy, marketplace, payment provider	Purchase, fulfilment, payment	Destination and ownership links
Data return	Platform and affiliated services	Reuse of interaction data, personalization	Usually limited transparency

These roles may be distributed across independent organizations or concentrated within one corporate ecosystem, and the business logics that large technology firms bring to health are themselves documented (45). Concentration is not evidence of improper steering, and integration can improve continuity and access. It does increase the organizational capacity to connect information provision with service designation, transaction and data return. This matters most when firms built on digital infrastructure move into health. Health is governed by different professional values, distributive principles and accountability expectations, and carrying one sphere's logic into another has itself been identified as a distinct risk (46). Control of health data and interoperability adds a further advantage, one that holds independently of any single recommendation (47). Synthetic gatekeeping concerns the conditions under which those organizational relationships become visible in the structure of an answer; it does not concern the moral character of a particular owner, and it does not license an inference of intent from corporate affiliation alone.

## 4 Cross-sector evidence and counterevidence

The shape of the evidence is itself telling. Look outside medicine and the picture sharpens: independent audits have already documented provider preference, self-promotion and framing effects that measurably alter what users choose. The numerical values reported in this section come from other domains and other research paradigms. They are reproduced to show that the mechanisms are measurable, not the magnitude to expect in health. Inside consumer health, the present search identified two studies examining commercial structure or repeated branded selection, and neither demonstrates systematic commercial steering. That distribution does not weaken the framework. It sharpens the question: whether mechanisms already observable elsewhere operate when the stakes are clinical, and at what magnitude (Table 4).

**Table 4:** Direct empirical evidence for commercial or provider-linked salience, by domain, among the sources identified in this synthesis.

Domain	Studies identified (*n*)	Principal finding	What transfers to health
Code generation	1 (8)	Systematic provider preference across seven models; silent substitution of a service the user specified	Mechanism; not stakes or effect size
AI-product recommendation	1 (49)	Owner-affiliated products ranked ∼0.2 positions above benchmark-justified level	Mechanism of dual-role conflict
Algorithmic hiring	1 (48)	Systems favoured options associated with their own provider (self-preferencing)	Mechanism of dual-role conflict
Consumer product recommendation and summarization	2 (50–51)	Marketing-style cues shift recommendations; summarization altered sentiment in 26.42% of cases and raised choice of the summarized product by 32%	Mechanism of framing and omission
Generative-engine visibility	1 (52)	Content optimization raised an experimental within-answer visibility metric by up to ∼40%	Mechanism of supply-side optimization
Consumer health answers	2 (58, 64)	One direct comparison found lower commercial bias than search; one vignette study recorded repeated branded selection without testing commercial causation	Not applicable: this is the domain in question

An audit of code generation covered seven models, six task categories and thirty scenarios. It found systematic provider preferences. In some cases the system quietly substituted a service the user had not asked for (8). Self-preferencing has since been documented in a further peer-reviewed setting, algorithmic hiring, where systems favoured options associated with their own provider (48). A second study compared model rankings of competing AI products against independent benchmarks and found owner-affiliated products ranked on average about 0.2 positions higher than performance justified — modest, but persistent after controlling for measured capability (49). Together these document the dual-role configuration in which the recommending entity participates in the recommended market.

In commerce, LLM recommendations respond to marketing-style cues such as social proof and authority framing (50). Synthesis is itself an intervention: models altered the sentiment of summarized content in 26.42% of cases, and readers of model-generated summaries were 32% more likely to choose the summarized product than readers of the source material (51). On the supply side, content can be engineered for visibility inside generated answers, with experimental within-answer visibility gains up to roughly 40% (52) — a measure of exposure, not of sales or traffic. Outside commerce, politically slanted conversational models shifted what participants said and decided (53). A separate agent, built to serve a corporate interest, did so by phrasing true content strategically (54). That is a demonstration of capability. It says nothing about how systems behave unprompted. Identity signals altered high-stakes recommendations without disclosure to users (55). Preprint evidence indicates that sponsored options can dominate conversational recommendation while labels fail to restore discrimination (56) and that models may favour platform interests under experimentally induced conflicts (57); both inform scenarios only.

Counterevidence constrains all of this. Effect sizes and user behaviour do not transfer across domains; a preference for a cloud service is not a preference for a therapy. Self-preferencing has not been found universally (22, 23). One study made the direct comparison. Answers about GLP-1 therapy were more objective and less commercially biased than conventional search results (58). It was exploratory, covering a single model family, one clinical topic and seventeen questions. Conversational systems are therefore not self-evidently worse than what they replace. The framework asks about the conditions of control and how far they can be inspected. It does not assert that today's health chatbots are more commercially biased than the search environments they partly replace.

The organizational-routing premise also has a health-specific analogue outside AI: physician–hospital vertical integration has been associated with shifts in downstream referral destinations and spending (59), showing that organizational structure can alter routing even when the informational layer is not generative. This does not establish an AI effect; it provides a health-domain comparator for the organizational-alignment hypothesis.

## 5 Medicine as a high-stakes test domain

Before turning to the health evidence, it is worth pausing on what we actually know — and what we do not — because the argument stands or falls on keeping those three categories apart (Table 5).

**Table 5:** What is known directly in health, what is known indirectly from other domains, and what is not known.

Status	Content	Permitted use in this article
Known directly in health (peer-reviewed)	Answer content is sensitive to system instructions, safety configuration, user framing, training data and drug-name form. One comparison found LLM answers less commercially biased than search; one vignette study recorded repeated branded selection.	Establishes configurability and motivates auditability. Does not support any claim about commercial use of configurability.
Known indirectly from other domains (peer-reviewed)	Provider preference in code generation; owner-affiliated ranking advantage in AI-product recommendation; framing and summarization effects on choice; supply-side optimization of within-answer visibility.	Establishes that the mechanisms exist and can be measured. Effect sizes are illustrative only and must not be read as expected magnitudes in health.
Documented architecture (company sources)	Consumer health assistants linking records, devices and third-party commercial services; an ecosystem linking primary care, prescription and pharmacy fulfilment.	Establishes architectural feasibility and the presence of commercial adjacency. Establishes nothing about routing behaviour.
Not known	Whether organizational configuration predicts the commercial structure of health answers; whether users detect such structure; whether disclosure changes their judgements; whether medicine differs empirically from other domains.	Stated as open questions and addressed in the research agenda, never as background assumptions.

Medicine is usually treated as a special case because harm potential, knowledge asymmetry, vulnerability and welfare-first expectations are higher than in consumer domains. We adopt that assumption to set scope, but it is empirical and not settled. The sparsity of direct health evidence, set against clearer demonstrations of provider preference elsewhere, makes health an unusually informative test domain. If organizational configuration leaves no reproducible trace in clinical answers once clinical confounders are addressed, the framework needs substantial revision. If it does leave one, the stakes are higher precisely because the same mechanisms have already been shown to operate in other markets. Whether commercial salience behaves differently in health than in matched non-health domains can be measured; Section 12 sets out the comparison.

Within health the evidence base is asymmetrical in a specific way: it documents configurability, not commercial use of configurability. Hidden system instructions converted customized general-purpose models into authoritative-sounding disinformation systems in 88 of 100 tested outputs (7). Safeguards vary between models and versions, making safety a property of deployment, not of the model (60). Models may follow a user's false premise despite holding the correct information (61). Small training-data manipulations alter medical behaviour while standard benchmarks fail to detect the change (6), and outputs are fragile to brand versus generic drug names (62). Under explicit purchase-intent queries, generative search has directed users toward specific medication sellers, including illegal pharmacies (63). In a 20-vignette obesity study, agreement between LLM medication choices and expert clinicians varied substantially across models, while one model produced hallucinations in 17 of 20 cases (64).

Two further health-domain findings make the unit-of-analysis problem concrete. In a randomized preregistered study of 1,298 members of the public, models performed strongly when tested alone, yet participants assisted by those same systems identified relevant conditions and appropriate dispositions no better than a control group; standard benchmarks and simulated users did not predict these interaction failures (65). Separately, an audit of generative search found that clinically appropriate answer text could coexist with links directing users to illegal online pharmacies, including a case in which the prose and the destination pointed in opposite safety directions (63). Neither study concerns commercial self-preferencing. Together they show that model capability, surface answer quality and the action pathway are separable objects of evaluation. These findings jointly establish that a medical answer is not the output of a model in isolation. It is produced by a system encompassing data, alignment, system instructions, retrieval, ranking, interface design, personalization, product policy and business model. Naming that system is not an accusation; it identifies the object that health-AI research should describe and audit.

Current product documentation and scholarly analysis show those same components being assembled in consumer offerings, and the detail matters (12, 14, 15, 66). A dedicated health experience launched in January 2026 links patient portals and wearable data to conversational answers, and its triage performance has already been evaluated in a structured test (67). It also permits connections to third-party commercial services: fitness, nutrition, weight management, grocery delivery. The provider reports that very large numbers of users ask health questions each week (12). A comparable healthcare offering from a second provider connects records, laboratory results and fitness data to a conversational interface (14). A third ecosystem brings primary care, virtual consultation, prescription of weight-management medication and pharmacy fulfilment into one corporate group, with prices shown in advance and same- or next-day delivery. Its documentation also states that clinicians send prescriptions to the pharmacy the patient chooses (15). Taken together, these sources establish architectural feasibility and the presence of commercial partnerships adjacent to health answers. They do not establish that routing is improper, and the documented preservation of external pharmacy choice is exactly the kind of boundary condition that evaluation must record.

## 6 What happens after the answer? content traces, pathway traces and auditability

Commercial visibility need not arrive through a single door. It could enter an answer through several pathways, and only one of them — the revenue pathway — requires a conversion or sponsorship incentive; the others can emerge from ordinary engineering and market structure. Outcomes divide into two families that must be measured separately. Content traces concern the answer text: naming, ordering, text share, strength of endorsement, omission, quality of comparison and responsiveness to a stated budget. Pathway traces concern what the answer makes possible: link destination, internal versus external booking, default pharmacy, purchase controls, friction, the presence of a no-purchase option and the data returned after the user acts. The two can diverge, as the pharmacy-link case shows (63). Table 6 sets out the pathways, and Figures 3A,B place them within the overall framework; two further qualifications are essential. First, the pathways are not mutually exclusive and can produce identical surface patterns. Second, they differ sharply in external identifiability: retrieval, ecosystem and revenue pathways leave observable traces in citations, links and routing, whereas training-data and alignment pathways cannot be distinguished from outside without provider cooperation. A taxonomy is not a causal model, and no audit conducted from the outside can attribute an observed pattern to a specific upstream pathway.

**Figure 3:**
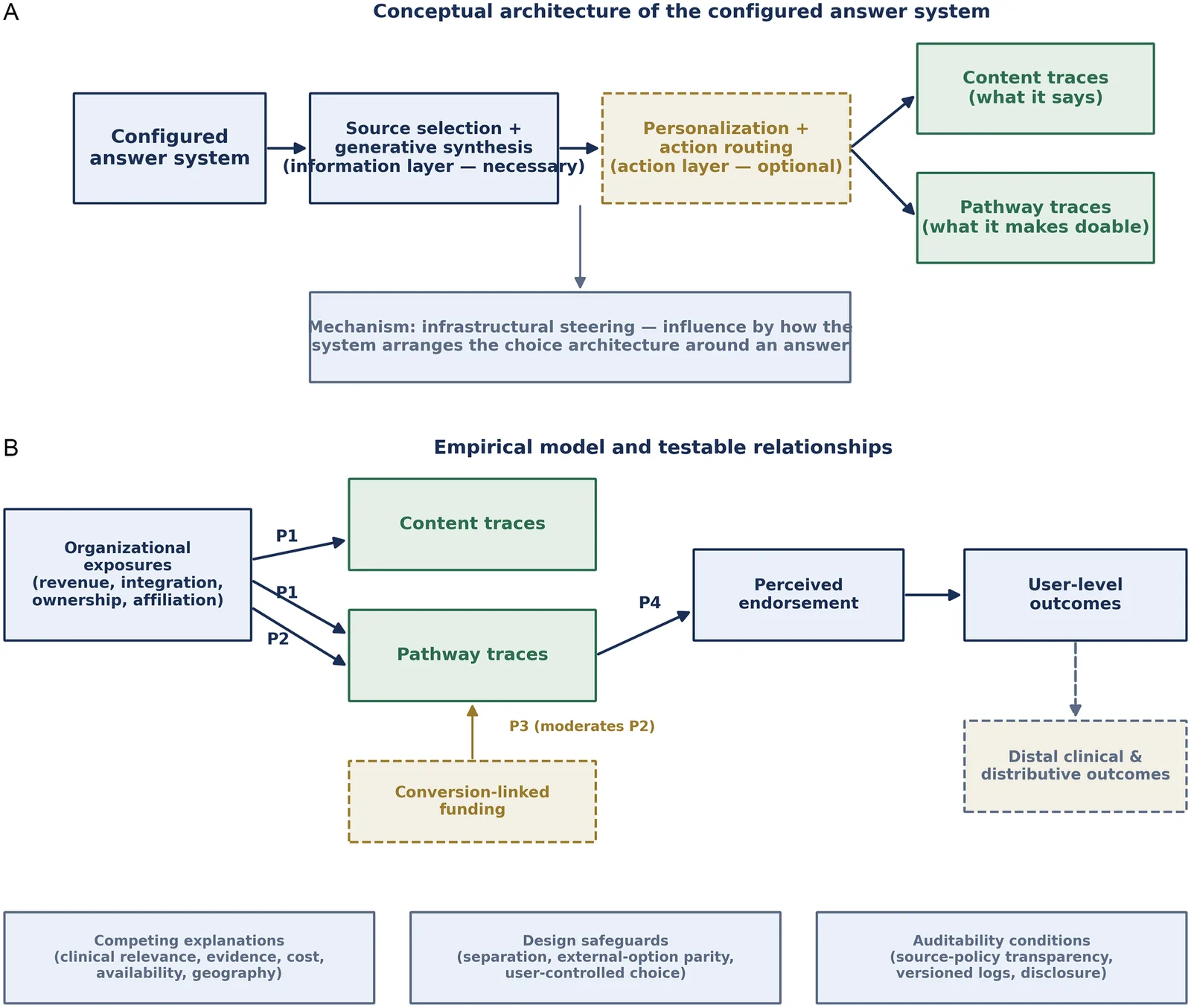
**(A)**. Conceptual architecture of the configured answer system. The configured answer system runs from source selection and generative synthesis (the necessary information layer), through optional personalization and action routing (the action layer), to two families of observable traces: content traces and pathway traces. Infrastructural steering is the mechanism. This panel shows what the construct is; it makes no causal claim. **(B)** Empirical model and testable relationships. Organizational exposures (revenue architecture, integration depth, concentration of control, ownership, affiliations) are hypothesized to shape content and pathway traces via P1 and P2; P3 conditionally moderates P2; P4 relates pathway traces to user-level outcomes through perceived endorsement; the measurement corollary M1 concerns surface–pathway divergence. Competing clinical and market explanations, and design safeguards and auditability conditions, are shown as side conditions that must be addressed before an organizational Reading is preferred. Distal clinical and distributive outcomes are dashed research horizons, not demonstrated endpoints. Arrows denote hypothesized relationships, not established causal effects.

**Table 6:** Pathways, evidence, alternative explanations, and external auditability.

Pathway	Mechanism	Alternative explanation	Externally auditable?	Evidence
Training-data salience	Entities with greater corpus prevalence, or strategically modified data, become more available to the model	Prevalence may reflect evidence, clinical use, or market share	No — requires provider access	(6, 62)
Retrieval salience	Indexed, highly ranked or optimized sources gain an additional route into the answer	Ranking can surface authoritative sources, not only commercial ones	Yes — via citations and links	(52, 63)
Alignment salience	Helpfulness and satisfaction objectives may favour concrete naming or acquiescence over friction	May reflect generic helpfulness errors, not commercial objectives	Partly — via behavioural probes only	(60, 61)
Ecosystem salience	An integrated provider can make its own adjacent service the easiest next step	Internal routing can improve continuity; external choice may remain formally available	Yes — via next-step routing	(8, 12, 14, 15, 49, 66)
Revenue salience	Advertising, affiliate or conversion incentives reward attention directed to paying or owned actors	Two of the four sources are preprints (56, 57); the others are commentaries. No direct health evidence	Yes — via disclosure and routing	(56, 57, 88, 89)

## 7 Trust, agency, and ethical stakes

Conversational design creates a relational experience. Anthropomorphic cues influence social presence, trust building and acceptance (30), and experimental work documents reliance on low-accuracy medical advice (68). Philosophical analysis identifies a reliability gap: patients and clinicians may be unable to verify the performance of an opaque system independently (69), motivating proposals for deliberately non-anthropomorphic interfaces (70). System quality nonetheless remains a leading determinant of acceptance, with oversight and certification contributing but not substituting for performance (71).

Clinical relationships may carry fiduciary or fiduciary-like duties, depending on jurisdiction and role; a general-purpose platform has no settled equivalent obligation. Information-fiduciary theory offers one governance model (72), while its critics warn that fiduciary language can obscure structural power and conflicted business models (73). We therefore do not posit a new construct here, and note only a possible divergence between the impression that a conversational interlocutor acts primarily for the user and the provider's actual obligations. Whether users attribute such obligations to health chatbots is an empirical question, listed in Section 12.

### 7.1 Ethical stakes

The concern developed here is not primarily one of market structure but of clinical ethics under conditions of privately governed information, and four principles are engaged. Respect for autonomy, understood relationally, not as isolated preference satisfaction (74), requires that a patient can identify the range of reasonable options and the interests attached to them; where synthesis conceals which alternatives were considered, autonomy is formally preserved while its epistemic preconditions are weakened. Non-maleficence is engaged because the designated next step carries clinical consequences: an unnecessary paid consultation delays care for some patients and imposes cost on others, and a preferentially surfaced medication is not a neutral suggestion when the user cannot evaluate the comparison. Beneficence cuts the other way and must be stated with equal force: integrated pathways can reduce fragmentation, shorten time to appropriate treatment and support adherence, and there is no ethical case against coordination as such. Justice is engaged by cost and access, and the distributive implications of consumer health assistants have begun to be examined directly (75): if commercial salience is insensitive to a patient's stated financial constraints, the burden falls on those least able to absorb it, and an architecture that is convenient for well-resourced users becomes a barrier for others. Four distinctions sharpen what is at stake. Epistemic autonomy concerns whether a person can find out why one option was shown and another was not; where that is unavailable, the capacity to evaluate advice is reduced even when the advice is sound. Relational autonomy locates choice in the informational conditions that surround it, so that a defensible answer delivered inside an architecture that hides its alternatives is not equivalent to the same answer delivered where alternatives are visible. Role morality distinguishes the obligations of a clinician, a platform, a search provider and a marketplace, which are not interchangeable and which users may not be able to tell apart when they arrive in a single conversational register. Procedural justice concerns whether a recommendation can be contested and its route reconstructed after the fact, and distributive justice whether routing varies with price, insurance, language or geography in ways that shift burden toward those least able to absorb it. These principles do not settle the question; they say what would have to be measured to settle it, and they locate the framework within the commercial determinants of health (76–79) and existing guidance on the governance of AI for health (80, 81) rather than within market regulation alone.

Autonomy requires more than the formal availability of a choice; it requires the epistemic conditions under which a choice can be examined — a visible range of reasonable alternatives, an intelligible basis for their ordering, and a route by which the recommendation can be contested. This connects individual autonomy to epistemic welfare: the social conditions and capabilities that let people obtain, interpret and challenge information relevant to their lives (82). At population level, repeated reliance on a small number of configured answer systems may concentrate interpretive authority and create correlated blind spots. The evidence does not establish long-term cognitive decline or systematic social manipulation, and those claims should not be made. The narrower concern is already serious: comparison, disagreement, a request for justification and a refusal to validate a false premise are the corrective friction through which judgement is tested. Where a fluent and relationally persuasive interface removes that friction while remaining organizationally opaque, an answer may be clinically plausible and still weaken the conditions under which autonomous judgement is formed.

## 8 Testable propositions and empirical implications

A framework earns its keep not by being proved on the spot but by making promises that others can later check. This one is not tested here; its value depends on whether it yields propositions that future research could support, constrain or overturn. Three core propositions and one conditional moderator hypothesis follow; they are stated as expectations about what would be observed, not as a study protocol. Four terms need conceptual definition first. An owner-linked option is a product, service or provider in which the operator of the answer system, its corporate group or a disclosed commercial partner holds an economic interest. A clinically reasonable comparator set is the group of options that current guidance, an independent expert panel or explicit eligibility criteria would treat as defensible for the presented indication, with efficacy, safety, eligibility, availability in the relevant jurisdiction, price and coverage recorded separately. The term is preferred to ‘clinically equivalent alternative’, because true equivalence is rare in medicine. For empirical tests, the comparator set should be specified before model outputs are coded and anchored to a jurisdiction-specific clinical reference standard; where expert adjudication is required, adjudicators should be independent of the organizational mapping used in P1. The counterfactual reference is the distribution of options a clinically appropriate answer to the same question would contain, judged against an independent clinical benchmark and not against another system's output. A routing difference counts as organizational advantage only where it persists after eligibility, availability, cost, coverage and geography are addressed; where it disappears under those controls, it is better described as justified integration. Justified integration is the limiting case in which an apparent internal–external pathway difference is accounted for by clinically or operationally relevant factors rather than organizational alignment; the practical corollary is that asymmetry is not itself the problem, and unexplained asymmetry is what an evaluation should investigate; Supplementary Table S5 sets out candidate audit criteria across ten domains, with the rule that residual asymmetry after these controls is an audit signal, not evidence of intent.

1. P1 — Organizational alignment. Holding clinical appropriateness and market availability constant, a documented change in organizational configuration is expected to change organizationally aligned salience in content or pathway traces. Organizational alignment must be specified before output coding by mapping ownership, affiliate and revenue relationships independently of the observed answer; where feasible, trace coding should be blinded to that mapping. Strong evidence would come from documented revenue or partnership changes, within-system comparisons or provider-access studies; cross-platform differences alone are insufficient, and where configuration cannot be documented the proposition is not testable.
2. P2 — Pathway asymmetry. As action integration deepens, the internal option is expected to gain a larger relative actionability advantage over clinically reasonable external options after accounting for the number of functions available in the interface. Support would require a reproducible increase in pre-specified pathway metrics such as visibility, number of steps, default status, prominence or completion friction; the mere presence of an internal service does not satisfy P2.
3. P3 — Conversion moderation (a conditional moderator of P2). The pathway asymmetry of P2 is expected to be larger where funding depends on conversion than where it does not. Because it requires revenue architecture and integration depth to vary independently — which a small, heterogeneous set of providers may not allow — this is offered as a conditional moderator hypothesis rather than a core, freestanding proposition, and is testable only where such independent variation exists.
4. P4 — Behavioural mediation. Holding clinical content constant, the effect of pathway asymmetry on choice is expected to be mediated by perceived endorsement: an asymmetric action architecture raises the perceived endorsement of the favoured option, which in turn shifts choice, willingness to pay and care-seeking. A direct effect on choice with no change in perceived endorsement, or a null indirect path, would disconfirm the mediation. This is the step at which an organizational trace would acquire welfare significance, and it is stated as a hypothesis to be tested.

Measurement corollary M1 (surface–pathway divergence): clinically comparable answer content can coexist with materially different action architectures, so evaluations restricted to prose underestimate organizational effects wherever links, defaults, routing, transaction conditions or data return differ. M1 is a measurement principle that P2 and P4 depend on, not an explanatory hypothesis in its own right. Box 1 sketches how it could be operationalized through paired content and pathway coding. P1–P4 and M1 stop at user-level outcomes; the clinical and distributive outcomes in Figure 3B — escalation, delay, cost, access, continuity, equity — are treated as distal research horizons downstream of these relationships, beyond what the present propositions test.

BOX 1 **Surface–pathway divergence coding framework (illustrative; not a validated instrument). The framework is intended as a set of candidate observables for future research and system audit, not as a validated scoring instrument or compliance checklist. It contrasts two audits of the same answer — what it says (content parity) and what it makes doable (pathway parity). Each component is coded separately for the internal, owner-linked option and for the clinically reasonable external option. A high-divergence pattern is the case shown in Figure 2: content that looks neutral above an action architecture that is not. No scalar weighting scheme is assumed here; future work may derive and validate a composite index that summarizes the difference between pathway and content divergence.**

**Table:** 

Content parity (what the answer says)	Pathway parity (what the answer makes doable)
option visibility; ordering; framing valence; comparator completeness	steps to each option; default selection; visual prominence; pre-filled payment or identity; internal-vs.-external accessibility; post-action data return

BOX 2 **A worked example (hypothetical; no real provider). Three configured systems answer the same obesity-medication question with the same clinically appropriate sentence — that several options exist and a clinician should be consulted — yet differ in what they make doable. A content-only audit would score them alike; a configured-system audit, using the coding framework (Box 1), separates them.**

**Table:** 

Configuration	What differs in the action architecture	Surface–pathway divergence
A — answer-only	options listed with links to independent guidance; no in-app booking or purchase	Low: content and pathway both diverse
B — integrated but justified	in-house consultation offered, external clinics shown with comparable prominence, affiliation disclosed, choice recorded	Moderate but explained: small, documented asymmetry
C — owner-linked asymmetry	identical prose, but one option carries a price, a pre-filled purchase and one-tap booking, while external providers require a separate search	High: content parity, low pathway parity

The example encodes the framework's central claim — that clinically comparable prose can sit above materially different action architectures — without asserting that any deployed system behaves like C.

The framework would be weakened if repeated studies found no reproducible link between organizational configuration and what the answer makes visible or easy to do — once clinically relevant explanations had been ruled out. Those studies would need to span providers, clinical domains, jurisdictions and product versions, and to account for evidence quality, safety, eligibility, availability, cost, coverage, geography, language and user intent. It would also require revision if integrated systems consistently preserved comparable visibility and accessibility of external options while improving continuity and patient outcomes, or if apparent effects failed replication across versions. Partial revision is equally informative: P2 holding without P1 would place the phenomenon in routing, not naming, and the reverse pattern would place it in naming, not routing.

### 8.1 From theory to health-chatbot evaluation

Table 7 sets out, for each proposition, the empirical contrast, predicted outcome and weakening condition. The propositions advanced here are not tested in this article. They are intended to guide future research examining whether organizational configuration leaves a reproducible trace in health-chatbot answers, and no single study design can establish such an effect. Cross-platform audits may identify recurring differences but cannot isolate revenue architecture or integration from model family, training corpus, retrieval implementation, alignment procedure and interface design, because providers differ simultaneously across many correlated dimensions and are few in number. Studies that vary an instruction within one system may establish whether answer structures are technically configurable, but an instruction describing a commercial role is not a revenue model, and a sentence cannot reproduce access to booking, pharmacy, payment, fulfilment and return data. Stronger evaluation will therefore require triangulation (Figure 4): longitudinal audits anchored to product versions, natural experiments accompanying documented product or partnership changes, documentary and regulatory analysis of actual revenue and integration arrangements, and, where obtainable, provider cooperation or supervised access to system instructions and routing objectives.

**Table 7:** Empirical contrasts, predicted outcomes and weakening conditions for P1–P4 and M1.

Proposition	Empirical contrast	Predicted outcome	Weakening or disconfirming result
P1 — Organizational alignment	A documented change in organizational configuration (revenue, partnership, integration), preferably within one system over time	Organizationally aligned salience shifts in content or pathway after clinical and market controls	No shift; a shift explained by clinical or market factors; or configuration that cannot be documented, in which case P1 is untestable
P2 — Pathway asymmetry	Answer only → outbound referral → internal booking → transaction and fulfilment → data return	The internal–external gap in visibility, steps, defaults and completion grows more than the number of functions	Comparable discoverability and friction for internal and external options across depths
P3 — Conversion moderation (conditional)	Pathway asymmetry under conversion-linked versus non-conversion funding, with the two exposures varied independently	Asymmetry is larger under conversion-linked funding	No moderation; effect present without conversion dependence; or exposures not independently identifiable
P4 — Behavioural mediation	Clinically comparable content paired with different pathways, shown to users	Perceived endorsement and choice differ with pathway at constant clinical content	Choice unchanged by pathway; or differences removed under external-option parity
M1 — Surface–pathway divergence (measurement corollary)	Clinically comparable prose paired with different links, defaults, routing or transaction conditions	Similar prose coexists with materially different destination, friction, completion or data return	Pathway equivalence whenever prose is comparable

**Figure 4:**
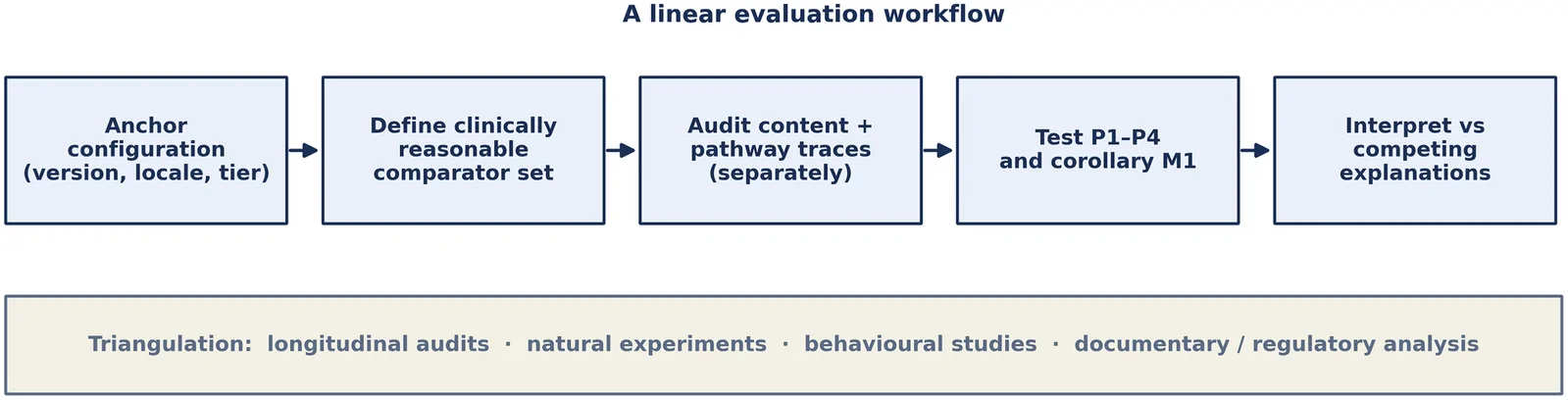
A linear evaluation workflow. Five steps in sequence: anchor the configuration (version, locale, account tier); define a clinically reasonable comparator set; audit content and pathway traces separately; test P1–P4 and the measurement corollary M1; and interpret against competing explanations. No single design suffices; the footer names four triangulating designs.

For implementers, the immediate implication is not to infer organizational steering from affiliation or integration, but to widen the audit boundary. A minimum evaluation should preserve the answer-producing configuration, assess content and pathway traces separately, define clinically reasonable comparators independently of the observed output, and document whether any internal–external difference remains after clinical appropriateness, availability, eligibility, cost and geography are considered. This does not constitute a validated audit protocol; it identifies the observable system properties that the present theory predicts will matter.

Salience is also not a single quantity. Evaluation should distinguish five outcomes — visibility (whether an option appears at all), ordering (where it appears), framing (how favourably it is characterized), omission (which clinically reasonable alternatives are absent), and routing (what the system makes easiest to do next) — because a provider may show little difference in factual content while still shaping the practical horizon of the user. Sentinel domains for such work might include treatment questions in obesity medicine, urgent-symptom routing, and over-the-counter medicines or supplements, each of which permits matched clinical comparison while exposing a different commercial pathway. Coding requires positive and negative cases, and two examples indicate the level of specificity needed. A branded medication named alongside its generic form, its class and a non-pharmacological option counts as visibility without ordering advantage; the same medication named first, described at greater length and followed by a link, with no generic or non-pharmacological option present, counts as ordering, framing and routing advantage together. A referral to the operator's own clinic counts as an owner-linked routing difference only where an equally appropriate external service exists, is available to the user in that jurisdiction, and is not shown; where no such service exists, the same output is coded as justified integration. A worked coding sheet with one positive and one negative example is provided as Supplementary Table S6.

Competing explanations must be addressed before any organizational reading is preferred: clinical appropriateness, evidence quality, safety, eligibility, availability, cost, coverage, geography, language, retrieval mode, brand familiarity, model family and product version. Where a difference disappears once these are included, it is better described as justified integration.

### 8.2 Version anchoring

Because deployed systems change without notice, any future evaluation of these propositions measures a moving target. Studies must anchor and report the product version, model identifier where exposed, retrieval mode, account tier, date and locale for every observation, and should be designed as repeated measurements, not single snapshots. A finding that a configuration effect was present in one release and absent in the next is a substantive result about governance instability, not a failed replication. Supplementary Table S7 provides a version-anchor template; where a version or model identifier is not disclosed, the field should record ‘not exposed by provider’ rather than being left blank.

## 9 Boundary conditions and strongest objections

The principal objections and replies are summarized in Table 8. What survives these objections is narrower than the claim that platforms control medical decision-making — and, precisely because it is narrower, harder to wave away: conversational systems concentrate partly opaque powers of selection, synthesis, personalization and action routing in organizationally governed interfaces; adjacent sectors show that such powers can produce provider preferences, framing effects and commercially consequential recommendations; whether comparable mechanisms operate in health is urgent, testable and unresolved.

**Table 8:** Objections, replies, and remaining areas of dispute.

Objection	Reply at the strongest defensible level	Status
Users retain agency; algorithmic governance is deterministic rhetoric.	Accepted that choices are not determined. The claim concerns the architecture of visibility, comparison, defaults and friction, not the elimination of choice (17, 19, 26).	Selection is established; magnitude of behavioural influence is context-dependent.
Search engines already gatekeep; the construct is a new label for an old process.	Gatekeeping is continuous, and generative gatekeeping has already been described (9–11). The proposed extension is the combination of source selection, synthesis, personalization and action routing within one conversational interface, examined in relation to organizational exposures such as ownership, revenue architecture and integration depth in a clinical setting.	Continuity established; whether the fusion warrants a distinct construct is legitimately contestable.
Brand mentions may reflect evidence or availability.	Commercial concern begins only where prominence cannot be explained by clinical relevance, evidence, safety, availability, cost or user need.	A brand mention alone is never evidence of promotion.
Steerability does not prove commercial steering.	Health studies establish configurability (6, 7, 60–62) but not its use for owner benefit; the framework states propositions about what would be observed, and about the conditions under which it would be seen.	Commercial steering in deployed health systems remains open.
Cross-sector results do not transfer to medicine.	Effect sizes and decisions do not transfer; mechanism hypotheses can be carried into health for independent testing, and a health-versus-other-domain comparison is listed in the research agenda.	Transferability is a research question, not an assumption.
Ownership predicts little on its own; providers are heterogeneous.	The framework is configurational: model owner, interface owner, deployer, retrieval, revenue, integration and market participation (Table 3). Because providers differ simultaneously across these dimensions and are few in number, no single design can separate them.	Which configurations predict salience is open.
Vertical integration can improve care.	Coordination benefits coexist with dual-role conflicts (22, 23). The question is whether alternatives remain visible and whether routing is clinically justified.	Net benefit is system- and context-specific.
Official product documentation is marketing material.	It is used here solely to establish the configuration a provider states it has deployed, never to establish performance, neutrality or intent; independent testing is required for behaviour and effects. Documented safeguards, such as an advertising-free health environment or preserved pharmacy choice, are recorded with the same status.	Architecture is documented; behaviour is not.
Fiduciary language may be paternalistic or ineffective.	Accepted as a risk (72, 73). Disclosure efficacy and attributed obligations must be measured; structural power cannot be reduced to labels.	The appropriate governance model remains contested.

## 10 Four configurations of medical information

Two questions, taken together, map the terrain: does a system's funding stand apart from conversion or depend on it, and is it a stand-alone answer interface or a deeply integrated gateway? Crossed, they yield four configurations (Figure 5). For this typology, ‘conversion-linked’ is analytical shorthand for funding or benefit structures in which completed downstream action creates a marginal organizational benefit; it is not intended to exhaust possible incentive architectures such as ownership margin, reimbursement, retention or cross-selling. These are configurations for analysis, not predictions, and engagement optimization operates as a cross-cutting dynamic within all four. The most consequential trajectory is not a chatbot displaying advertisements; it is the gradual disappearance of boundaries between information, recommendation, service provision, transaction and data return inside one interface. Product documentation and scholarly analysis show components of that pathway already assembled (12, 14, 15, 66), which demonstrates architectural feasibility; the same documentation records that patients may choose an external pharmacy (15). A public or subscription system may still reflect political, institutional or data biases (83, 84).

**Figure 5:**
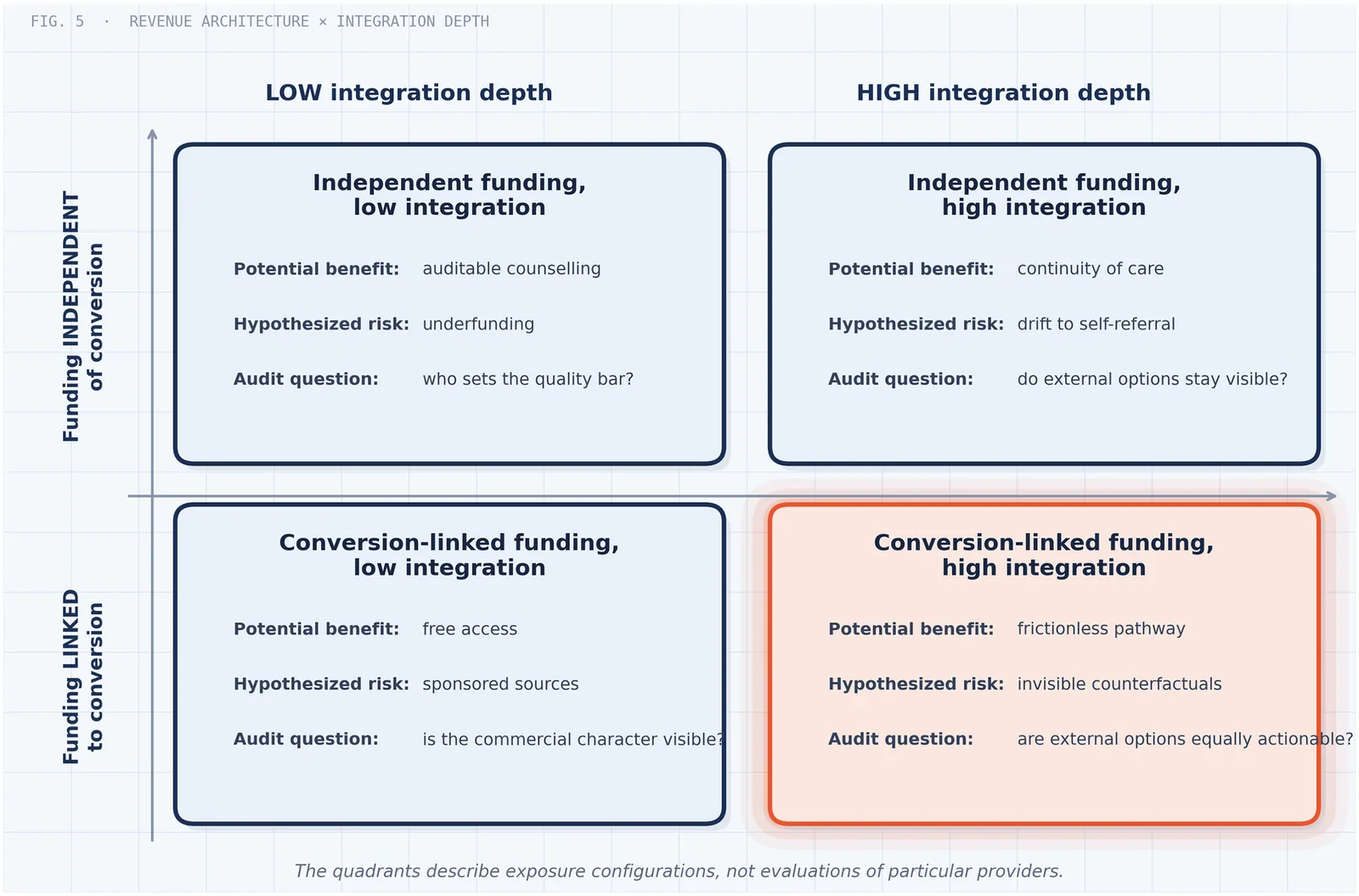
Revenue architecture   ×  integration depth. The quadrants name exposure configurations, not evaluations of particular providers; each carries potential benefits as well as hypothesized risks, and the audit question in each quadrant identifies the principal feature to test. User vulnerability is modelled as an effect modifier rather than an exposure dimension: the same configuration may impose different burdens depending on digital and health literacy, resources, language and access constraints (Supplementary Table S8).

### 10.1 From configurations to trajectories: a bounded foresight lens

The four configurations are analytical types, not forecasts, but they help chart how health-information systems might evolve. Using 2030 only as an illustrative horizon, and following standard scenario practice — internally consistent alternative futures rather than a single prediction (85) — we treat the trajectories as plural and reversible: systems may move between configurations as regulation, business models, interoperability, public infrastructure and user expectations change. Several drivers look consequential: agentic systems that can complete actions; interoperable health and transaction data; advertising or conversion-based business models; expansion of platform-owned health services; rules on self-preferencing and disclosure; and the strength of public, clinical and nonprofit alternatives. These may reinforce or offset one another, and the point is not to predict a winner but to name the early signals of movement and the governance questions each raises before a configuration hardens (Table 9).

**Table 9:** Illustrative trajectories to a 2030 horizon, with early signals and the governance question each raises.

Trajectory	Character	Early signals of movement	Principal governance question
Independent funding, low integration	Information separated from conversion and transaction	Public models; ad-free subscription; interoperable sources	Who sets the quality standard and the admissible source range?
Conversion-linked funding, low integration	Answer interface dependent on advertising or conversion	Sponsored recommendations; affiliate links; paid visibility	Can users recognize the commercial character of selection?
Independent funding, high integration	Deep integration with funding independent of conversion	Booking, e-prescription and referral inside public or clinical systems	Does integration improve access without narrowing choice?
Conversion-linked funding, high integration	Answer, recommendation, purchase and data return in one ecosystem	Default own-services; in-house pharmacy, booking and fulfilment	Do external alternatives remain equally visible and accessible?

## 11 A graduated governance and accountability response

The ladder is not proposed as a compliance standard. It derives governance intensity from the evidentiary structure of the theory: stronger interventions require stronger evidence that organizational configuration produces persistent, welfare-relevant asymmetry. Because present evidence establishes capability, opacity and adjacent-sector precedent, not documented systematic harm in medical answers, governance should be proportionate and triggered by observable risk. Accountability requires an identifiable actor, a forum, a standard and the possibility of consequence (86); for health chatbots, none of the four is currently settled, and legal scholarship has begun to map how such systems fall between existing European regimes (87). The rungs fall into three evidentiary tiers (Table 10). Disclosure, auditability and independent comparative testing are warranted now, by opacity alone. Comparative duty and separation of sponsorship require a demonstrated pattern of organizationally aligned salience. Structural remedies require, in addition, evidence of clinical or market harm that lighter measures have failed to prevent. A trigger is stated for each rung above the first tier.

**Table 10:** Evidence-to-governance mapping: what evidence triggers each rung.

Rung	Governance action	Evidentiary trigger
1	Basic disclosure	Opacity or configuration complexity alone; no proposition need be confirmed
2	Auditability (versioned logs, configuration disclosure)	Inability to reconstruct the configuration retrospectively; no proposition required
3	Independent comparative testing	A standing risk configuration warranting systematic audit; not yet evidence for P1 or P2
4	Comparative duty in high-stakes answers	Replicated P2 (pathway asymmetry) or failure of external-option parity, especially where P1 strengthens organizational attribution
5	Separation of sponsorship from generation	Repeated P3 support: asymmetry intensifies under conversion-linked funding, or sponsorship demonstrably affects source selection or routing
6	Structural remedies	Replicated organizationally aligned asymmetry plus demonstrated welfare, clinical or market harm (ideally via P4 or downstream outcomes), plus failure of lighter measures

The response is graduated across six rungs, each triggered by a defined evidentiary threshold (Figure 6): basic disclosure and auditability are warranted by opacity alone; independent comparative testing draws on established audit designs (88, 89); and comparative duty in high-stakes answers, separation of sponsorship from generation, and structural remedies each require progressively stronger evidence of organizationally aligned salience or of harm that lighter measures have failed to prevent.

**Figure 6:**
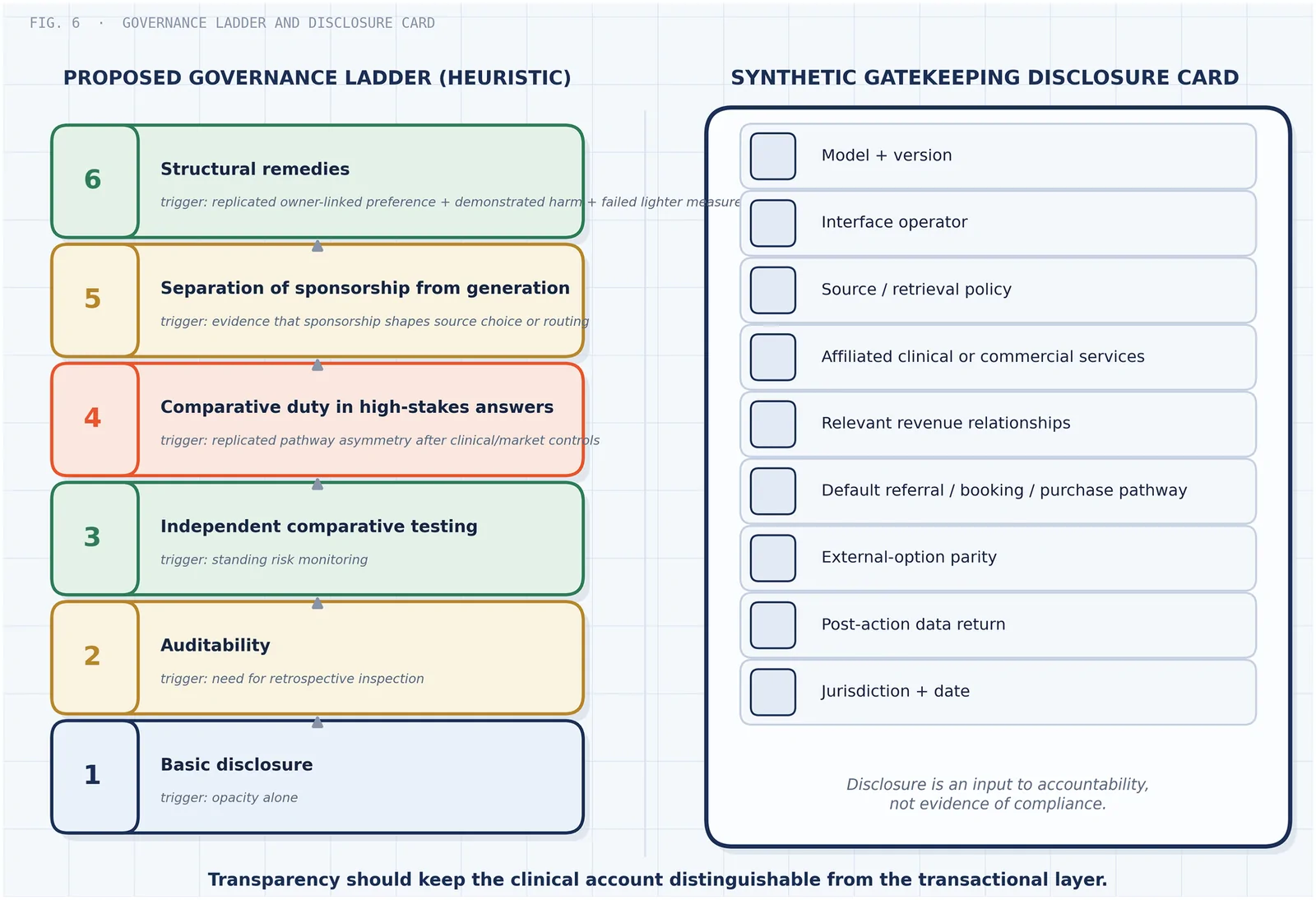
A proposed, heuristic governance ladder (not an existing regulatory standard), with a synthetic gatekeeping disclosure card. The ladder escalates from basic disclosure (triggered by opacity alone) to structural remedies (triggered only by replicated owner-linked preference together with demonstrated clinical or market harm and the failure of lighter measures). The disclosure card is a version-specific minimum reporting standard for a configured answer system; disclosure is an input to accountability, not evidence of compliance.

Rungs 1–3 are governance under uncertainty; rungs 4–6 are governance in response to evidence. The mapping specifies procedural and measurable triggers — replicated, persists after clinical controls, requires a separate search, a difference in the number of steps — but does not assign universal effect-size cut-offs, because the current literature provides no empirical basis for a single clinically meaningful threshold across domains.

### 11.1 Organizational conflict-of-interest disclosure

Provider identification alone is insufficient when a health answer can route the user toward a service supplied by the platform, its corporate group or a commercial partner. At the point of recommendation, the interface should disclose material ownership, affiliate, sponsorship or revenue relationships; identify whether the suggested pathway is internal or external; and state whether clinically reasonable external and no-purchase alternatives were considered. Where source selection or routing is optimized partly for conversion, that objective should be disclosed in language proportionate to the user's decision context. Such organizational conflict-of-interest disclosure is a functional transparency requirement, not an assertion that every platform owes a legally established fiduciary duty. The generated clinical account should remain distinguishable from the transactional layer, external-option parity should be testable, and versioned logs should permit independent assessment of source selection, omission, prominence and routing.

### 11.2 A synthetic gatekeeping disclosure card

Disclosure should follow structural safeguards, not replace them. Where a health assistant can route a user toward a service, product or transaction connected to its provider, the clinical account should remain distinguishable from the transactional layer, clinically reasonable external and no-purchase alternatives should receive comparable visibility, and the default pathway should be independently testable. A disclosed conflict that continues to shape choice is not resolved by a label; referral pathways from conversational systems already carry measurable economic consequence (90). Organizational transparency should not be reduced to a generic statement that an answer was generated by artificial intelligence. A meaningful disclosure should describe the organizational configuration responsible for producing and operationalizing the answer. We therefore propose a synthetic gatekeeping disclosure card: a standardized, version-specific description of the actors, relationships and action pathways that may shape a health-chatbot response. The card would record the model developer and model version; the interface operator; the retrieval and source-selection policy; affiliated clinical, pharmacy or commercial services; revenue relationships relevant to naming or routing; default booking, purchasing or referral pathways; whether external alternatives are displayed with comparable visibility; what data return to the platform after the user acts; and the jurisdiction and date of the configuration described.

Existing model and system documentation typically describes technical capabilities, limitations and safety evaluation. The proposed card does something different: it maps the organizational chain behind a specific health-answer configuration, including revenue relationships, action-routing defaults, external-option parity and post-action data return. The card would not determine whether a system is biased and should not be presented as a substitute for independent auditing. Its purpose is to make the configuration inspectable, and to keep organizational transparency separate from claims about intent. A provider could disclose deep integration while demonstrating external-option parity; conversely, the absence of formal ownership would not exclude commercial relationships capable of shaping visibility. Disclosure would therefore serve as an input to accountability, not as evidence of compliance, and its fields map directly onto the layers set out in Table 3.

Disclosure is not assumed to solve the problem. Chatbot disclosure demonstrably alters consumer behaviour (91), while emerging experiments suggest labels may not restore discrimination (56). Governance evaluation must therefore measure outcomes, not the presence of labels.

### 11.3 Existing regulatory interfaces and enforcement pathways

The ladder does not sit in a legal vacuum, though no current instrument maps onto it exactly. Several regimes provide partial hooks. The EU Artificial Intelligence Act imposes transparency obligations on systems that interact directly with natural persons (Article 50), but establishes no general duty to disclose the ownership or revenue architecture proposed here (92). The Digital Markets Act prohibits designated gatekeepers from more favourably ranking their own products or services [Article 6 (5)] — a close conceptual analogue for pathway asymmetry — yet its scope is limited to designated gatekeepers and core platform services (20). The General Data Protection Regulation governs the personal data generated and returned when a user acts on an answer, but not commercial salience as such (93). Where a tool meets the definition of a regulated medical device, lifecycle assurance and change-control expectations attach: the U.S. Food and Drug Administration has issued final guidance on predetermined change control plans for AI-enabled device software functions, a direct precedent for configuration and change assurance, conditional on device status (94). WHO guidance supplies a global normative benchmark — transparency, accountability, autonomy and equity among its principles for governing AI for health — but is soft law without its own enforcement mechanism (80, 81). No single regime obliges the disclosure card proposed here; the contribution is to show where each could attach and where the gaps lie (Table 11).

**Table 11:** Existing regulatory interfaces, potential application, and limits.

Regime	Relevant hook	Potential application	Principal limitation
EU AI Act	AI-interaction transparency (Art. 50)	Basis for disclosing AI mediation to users	Does not cover organizational ownership or revenue transparency
Digital Markets Act	Self-preferencing in ranking [Art. 6 (5)]	Strong conceptual comparator for pathway asymmetry	Only designated gatekeepers and core platform services
GDPR	Personal data, profiling and data return	Applies where the pathway generates and returns personal data	Does not regulate commercial salience itself
Medical-device regulation (e.g., FDA PCCP)	Lifecycle assurance and change control	Configuration and change assurance for regulated tools	Applies only where the tool is a regulated device
WHO AI-for-health guidance	Transparency, accountability, autonomy, equity	Global normative benchmark for governance	Soft law; no independent enforcement mechanism

## 12. Research agenda

The agenda is organized into four streams — measurement, causal and behavioural testing, equity and co-design, and governance evaluation.

1. Documentary and longitudinal work on organizational configuration: characterize actual revenue arrangements, partnerships and service integrations of consumer health assistants, and track answer structure across documented product changes, not at one point in time.
2. Cross-platform descriptive audits: characterize the current landscape with full version anchoring, treating results as hypothesis-generating, not as tests of organizational effects. Obesity medicine is a suitable starting domain because it is a clinically well-characterized area with guideline benchmarks against which necessity can be judged (95, 96), and because commercial products and programmes in this domain are numerous.
3. Domain comparison: run matched audits in health and in at least one non-health domain where provider preference has already been documented, such as cloud-service or AI-product recommendation, using identical prompt structures and outcome measures (visibility, ordering, framing, omission, routing). This tests directly whether medicine is empirically the critical case.
4. Health-adjacent self-preferencing replication: adapt provider-preference designs (8, 49) to telehealth providers, pharmacies and health applications.
5. Behavioural studies: randomize answers matched for clinical content and appropriateness, differing only in commercial salience and measure perceived endorsement, trust, choice, willingness to pay, and whether users can detect that a recommendation is commercially structured.
6. Equity and co-design: co-design audit scenarios, comparators and disclosure formats with people who differ in digital and health literacy, language, disability and accessibility needs, and socioeconomic resources, affordability and insurance status, so that vulnerability is examined as an effect modifier of configuration rather than a separate typolog*y* axis (Supplementary Table S8).
7. Attributed obligations and disclosure efficacy: measure what duties users believe a health chatbot owes them, and which disclosure formats, if any, restore discrimination (56, 71, 91).
8. Counterevidence tracking: report null results, refusal behaviour and findings of lower commercial bias than incumbent search (58) with the same prominence as positive findings.
9. Governance evaluation: treat each rung of Section 11 as an intervention with measurable outcomes, not as a normative recommendation.
10. Population-level diversity audit: run the same clinically benchmarked scenarios across platforms, user profiles and repeated sessions, and measure diversity of clinically reasonable comparator sets, correlation of omissions, concentration of routing destinations and stability across versions.
11. Longitudinal framing study: examine whether repeated interaction narrows or broadens the set of alternatives users generate on their own, and whether personalization, warmth and disagreement behaviour predict changes in confidence, verification and care-seeking.

## 13 Discussion

### 13.1 Theoretical contribution

The contribution claimed here is not the construct name, which the article does not treat as its own. What is offered is a theory of observable organizational traces in configured health answer systems: an account of where an organizational effect would enter, what it would leave behind in content and in pathway, and what would show that it had not. Its core is a change of unit. Evaluation has focused on the language model; we propose the configured answer system instead. Nobody encounters training data or model weights. What reaches a patient has passed through system instructions, retrieval and ranking, source policies, personalization, interface design, safety rules, and whatever ownership and service relationships the operator has entered into. Accuracy at the model level remains necessary. It does not tell us why one option became visible and another did not. The account extends work describing generative search chatbots as meta-gatekeepers (11) and the emerging formulation of LLMs as synthetic gatekeepers in the public sphere (37); its distinct move is to connect generative mediation to a health-specific causal and ethical framework in which revenue architecture and integration depth are the organizational predictors, content and pathway traces the observable outcomes, and clinical relevance, affordability, availability and safety the specified competing explanations.

Counterevidence is constitutive, not exceptional. The relative abundance of mechanism evidence outside medicine, and its near-absence inside it, is part of that same picture. The direct comparison of common GLP-1 questions found LLM answers more objective and reproducible than conventional internet search (58). Synthetic gatekeeping is therefore not the claim that generative systems are necessarily more commercially biased than search engines; it identifies a redistribution and concentration of mediating functions and asks when that configuration produces a reproducible organizational trace. A system may exercise synthetic gatekeeping while improving information quality. The normative concern intensifies only where opacity, welfare stakes and conflicted routing combine. The comparative question is therefore not whether conversational systems are more biased than search, but under which configurations either system preserves clinically reasonable alternatives and makes its mediation inspectable.

### 13.2 Ethical significance in health

Accuracy alone does not exhaust ethical evaluation. A medically accurate answer may remain ethically problematic if its framing, ordering, omissions or action pathways systematically privilege options aligned with the provider's commercial position. Harm can arise through delayed escalation, unnecessary purchasing, narrowed comparison, or omission of lower-cost and non-commercial options; conversely, integrated systems may reduce fragmentation, simplify access and improve continuity when implemented with appropriate controls (83, 84). Integration's value turns on performance, on who benefits, on whether alternatives survive, and on who answers for downstream effects. At population level, the commercial determinants framework shows how private-sector structures shape exposure, access and outcomes (76, 79), and when information environments are themselves treated as determinants of health (77), source visibility and routing become distributive questions rather than matters of interface preference. Platform-configured answers may interact with affordability, insurance status, language, geography and digital literacy, steering different users toward different practical horizons. That is why the analysis belongs to a public-health ethics perspective on the role of large technology firms in medicine (97), and to existing guidance on governing AI for health (80, 81).

### 13.3 Societal consequences: personalized interfaces, correlated blind spots

Earlier platforms mainly personalized exposure. Conversational systems can additionally personalize interpretation, reassurance and action — a shift from visible ranking towards synthesized authority. A results page showed several domains and left at least part of their disagreement on view; a conversational system may speak in one relational voice, tailor its reasoning to the user, and attach a route to consultation, purchase or service delivery. The distinction is not absolute: search was never neutral, and conversational systems can display sources and alternatives. It does, however, change auditability. Influence becomes individualized, transient and hard to observe as a common public message.

Two feedback loops deserve empirical attention. In a cognitive–affective loop, the user brings a premise, a fear or a preferred reading; the system answers within that frame; the answer raises trust or conviction; and the next questions are asked from a narrower starting point. In an action–data loop, the answer routes the user toward a service or transaction; the action generates new data; and the next round of personalization becomes more precise. Neither loop demonstrates manipulation. Both name pathways through which a configured system may help produce the very preferences and problem definitions to which it then adapts.

The population-level question is therefore not exhausted by whether any single answer is biased. It is also whether a small number of systems systematically narrow the range of interpretations and pathways available across many users. The risk is not necessarily one uniform message imposed on everyone; it may be personalized conformity — answers that feel individually tailored at the surface while reproducing similar categories, omissions and action architectures underneath. Evidence of generative monoculture makes the mechanism plausible, but its operation in health remains untested (33). Evaluation should accordingly measure cross-user output diversity, correlation of omissions, concentration of routing destinations, and whether independent systems preserve genuinely distinct clinically reasonable comparator sets.

### 13.4 Limitations

Four limitations constrain the claims beyond the methodological boundaries already stated in Sections 8.1 and 8.2. First, the evidence base is heterogeneous, and direct health evidence on owner-linked or revenue-linked steering is sparse. A targeted narrative synthesis can establish coherence, mechanism plausibility, counterexamples and testable predictions, but it cannot estimate prevalence or causal effect. Findings from code generation, product recommendation, political persuasion and platform competition generate hypotheses for health rather than proof about it. Second, affiliated services may be more available, cheaper, better integrated or better supported by evidence, and low output diversity may reflect model behaviour, not commercial preference. Third, access to system instructions, routing objectives and internal logs is unavailable to independent researchers, so audits can identify reproducible patterns but organizational cooperation or regulatory access is required to explain how those patterns arise. Fourth, synthetic gatekeeping is not confined to large commercial platforms. Public, clinical, insurer and nonprofit systems also select sources, encode priorities and structure action. We emphasize multi-market platforms because they combine infrastructure, distribution, data and adjacent services at scale, not because mediation is absent elsewhere. The framework deliberately overlaps with gatekeeping, platform governance, choice architecture and self-preferencing, and should be judged by whether its health-specific synthesis yields clearer measurements and falsifiable comparisons, not by terminological novelty.

### 13.5 Implications for health-AI evaluation

Evaluation should report the answer-producing configuration, not only the model name: provider, model and interface version, date, jurisdiction, account state, retrieval or browsing setting, visible sources, links, action buttons, and any integration through which a user can book, purchase, message or submit data. The same reorientation — from validating the model to assessing the AI-enabled workflow and its site-level governance — is being argued in adjacent clinical-AI settings (98).

For system developers and deployers, this shifts assurance from model validation alone toward configuration assurance: a clinically safe model can participate in materially different interventions depending on retrieval, interface, routing and service integration, so these layers should be versioned and auditable alongside model performance, and the disclosure card of Section 11.2 provides a template for what such assurance records.

For independent evaluators, the corresponding unit is the observable configured system — not only what answer was produced, but which alternatives were visible, which were actionable, how much friction separated them, and where action and data subsequently travelled. Commercial-salience audits should preregister paired prompts, use independent clinical reference standards, blind outcome coding, distinguish visibility from routing, and repeat sentinel scenarios across versions (Box 3).

BOX 3 **What an external audit can and cannot establish. Cross-sectional audits can identify reproducible, organizationally aligned patterns in what users are shown and what they can do next; they cannot by themselves establish the upstream mechanism, commercial intent, or the direction of causation.**

**Table:** 

Externally observable (no provider access)	Requires provider or regulatory access
Option visibility, ordering and framing; links and citations; default selection; action friction and number of steps; internal-versus-external routing and destination; external-option parity; post-action data flows visible to the user	System instructions and answer rules; optimization and reward objectives; revenue-sensitive routing logic; internal retrieval weighting; training-data composition; logs sufficient to attribute a pattern to a specific upstream pathway or to intent

For regulators, the central question is not whether a provider has an incentive in the abstract, but whether that incentive leaves a reproducible trace in what users are shown, how alternatives are characterized, and what the system makes easiest to do; Section 11.3 maps where existing regimes could attach to that question.

## 14 Conclusion

Who speaks when a health chatbot answers? No single actor does. The answer emerges from a governance chain distributed across model developers, interface operators, retrieval providers, deployers and, in integrated systems, clinical, transactional and data-service partners — an arrangement the person reading the answer can only partly see, and not a neutral repository of medical knowledge nor a model working alone. Nothing assembled here shows that a platform bends medical answers for profit; the one head-to-head comparison we found pointed, if anything, the other way. The claim is narrower, and we think more durable. The architecture that would make such steering possible already exists and is documented. Provider preference has been measured in neighbouring markets. And the answer format hides much of its own machinery from the person it is advising, while evaluation practice still treats the model, rather than the configured system, as the thing to be checked. That combination does not prove that clinically meaningful steering is happening. It is enough to justify looking — carefully, comparatively, and soon. The real question is not whether the platforms have already captured medical decision-making. It is whether we will keep the means to inspect and contest the infrastructures through which medical reality is selected and explained, while the line between clinical relevance and organizational interest is still ours to see. Two health chatbots can say the same thing and still create different choices; that is why the unit of evaluation is not the model output alone, but the configured answer system in which information becomes action.

Earlier platforms largely personalized what people saw. Configured answer systems can also personalize how uncertainty is interpreted and what becomes easiest to do. The societal question is therefore not only whether a chatbot gives a biased answer, but whether a small number of privately governed systems become the infrastructure through which millions of people define their health problems, resolve their doubts and move from information to action. Keeping the ability to inspect, compare and contest that infrastructure is a condition of autonomy — not an optional transparency feature.

## Data Availability

The original contributions presented in the study are included in the article/Supplementary Material, further inquiries can be directed to the corresponding author.

## Supplementary material

SUPPLEMENTARY MATERIAL Comprises eight sheets. S1 assigns one mutually exclusive evidence stream, peer-review status and permitted inference to every numbered source; S2 reproduces the counts reported in Section 2; S3 provides the retained search log; and S4 lists the prepared but unexecuted strings. Four sheets added at revision operationalize the framework: S5, audit criteria for justified integration (candidate decision rules); S6, a sample content-versus-pathway coding sheet with worked positive and negative examples; S7, a version-anchor template to be recorded for every observation; and S8, a vulnerability-by-configuration matrix in which user vulnerability is modelled as an effect modifier.
